# Association of ambient temperature and influenza-like illness with acute appendicitis: an ecological study using 22-year data

**DOI:** 10.1186/s12889-025-22318-x

**Published:** 2025-03-29

**Authors:** On Tai Ken Yu, Xiaoting Jiang, Conglu Li, Yawen Wang, Yuchen Wei, Ka Chun Chong

**Affiliations:** 1 Jockey Club School of Public Health and Primary Care, The Chinese University of Hong Kong, Hong Kong Special Administrative Region, China; 2 Division of Landscape Architecture, Department of Architecture, Faculty of Architecture, The University of Hong Kong, Hong Kong Special Administrative Region, China; 3 Clinical Trials and Biostatistics Laboratory, Shenzhen Research Institute, The Chinese University of Hong Kong, Shenzhen, China; 4 Centre for Health Systems and Policy Research, The Chinese University of Hong Kong, Hong Kong Special Administrative Region, China

**Keywords:** Time series, Acute appendicitis, Temperature, Influenza

## Abstract

**Background:**

While acute appendicitis poses a significant disease burden worldwide, its etiology is not completely known. Previous studies have separately demonstrated its associations with ambient temperature and seasonal influenza, but there was no study that examined two exposures concurrently, leaving room for confounding and failing to isolate the effects of these two factors. This study aims to quantify such associations under a unified model, using population-level data in Hong Kong from 1998 to 2019.

**Methods:**

The study outcome of weekly acute appendicitis admissions was analyzed with a number of covariates. The major covariates of interest included weekly mean temperature and three strain-specific influenza-like illness-positive (ILI+) rates, which were proxies for the activities of the respective influenza strains. Other covariates including weekly mean relative humidity, total rainfall and a composite index for air pollution were used for confounder control. A generalized additive model under the framework of distributed-lag non-linear model and quasi-Poisson distribution was used for multivariate analysis.

**Results:**

A significant positive association between ambient temperature and acute appendicitis admission was found, with a cumulative adjusted relative risk (ARR) of 1.082 (95% CI: 1.065–1.099) comparing the 95th percentile to the median temperature. ILI + rates for influenza A/H1N1 and A/H3N2 were found to significantly and negatively associate with acute appendicitis admission, with cumulative ARRs of 0.961 (95% CI: 0.934–0.989) and 0.961 (95% CI: 0.929–0.993) respectively, comparing the 95th percentiles to zero. No significant association was found between ILI + rate for influenza B and acute appendicitis admission.

**Conclusions:**

While high temperature was associated with acute appendicitis admission, a negative association of influenza infection was showed. The mechanisms underlying the above associations should be investigated in future studies, with the aim of formulating preventive strategies against acute appendicitis that take environmental exposures into consideration.

**Supplementary Information:**

The online version contains supplementary material available at 10.1186/s12889-025-22318-x.

## Background

Appendicitis is a common surgical condition both internationally and in Hong Kong. Its global incidence in 2019 was estimated to be 229.9 per 100,000, with the high-income Asia Pacific region having the second highest age-standardized incidence of 448.1 per 100,000 [1]. While uncomplicated acute appendicitis, if treated promptly, has a low mortality rate of less than 0.1%, the rate is significantly higher when complications occur [2]. For instance, a gangrenous non-perforated appendicitis has a mortality rate of 0.6%, while an outright perforated appendicitis can lead to a mortality of as high as 5%.^2^ In 2019, the global age-standardized years-lived-with-disability (YLDs) attributable to appendicitis were 2.7, representing a 20.4% increase from 1990 [1]. It was estimated that, in the United States alone, the cost of hospitalization associated with appendicitis was up to 3 billion US dollars per year [3]. 

While being a common condition with a significant disease burden, the exact etiology of appendicitis remains unclear to date. Past literature hinted that the condition is likely the result of the interplay among infectious agents, genotypes and environmental factors, such as seasonality [4]. Furthermore, while bacterial infection is known to directly contribute to the inflammatory process, some studies have suggested the possible involvement of viruses through various mechanisms [5]. 

There have been studies examining the relationship between meteorological factors and the incidence of appendicitis. Two observational studies in Mainland China and Taiwan respectively demonstrated that hospitalization for acute appendicitis were significantly associated with ambient temperature, after controlling for seasonal trends [6, 7]. Outside of Asia, a cohort study utilizing insurance data from the United States also suggested a positive association between temperature and acute appendicitis, independent of seasonality [8]. However, in all of the above studies, only meteorological factors were considered in their statistical analyses, and the potential effect of influenza activity was neither mentioned nor controlled for.

Regarding the association between influenza infection and acute appendicitis, a study in the United States reviewed the data from the National Hospital Discharge Survey from 1970 to 2006, and performed cointegration analysis of time series data [9]. It was found that the annual rates of influenza infection and non-perforated appendicitis displayed a similar trend over time, while the trend for perforated appendicitis was significantly different [9]. The authors postulated that influenza infection may predispose to appendicitis through mucosal ulceration or hyperplasia of lymphoid tissues in the intestines, providing a favorable condition for secondary bacterial infection [9]. However, meteorological factors were not controlled for in the study. Moreover, no similar study was found using data from a smaller time frame, such as weekly influenza rates. Since weekly or seasonal variations in influenza rates and acute appendicitis admissions were averaged out in annual data, any potential association in these smaller time scales would not be captured.

Previous studies either examined the effect of ambient temperature or that of influenza infection on acute appendicitis, without considering the two factors concurrently. This may result in confounding effect, since influenza activity and meteorological factors (including temperature) are highly associated [10]. 

In this study, we aim to concurrently examine the association between ambient temperature as well as influenza infection and the incidence of acute appendicitis in Hong Kong, using multivariate modeling that includes a range of meteorological and environmental exposures. This can address the issue of confounding and allow the isolation of independent effects of ambient temperature and influenza infection.

In addition, Hong Kong usually has two small peaks of seasonal influenza activity annually, during summer and winter time respectively [10]. This is different from many other regions where there is only a single peak in winter. In other words, using influenza activity and meteorological data in Hong Kong has the benefit of avoiding the problem of multicollinearity, where temperature is strongly negatively correlated with influenza infection rates, on top of the abovementioned research gap in literature.

## Methods

### Data

The weekly numbers of incidence cases of acute appendicitis presented to public hospitals in Hong Kong in the period of January 1, 1998 to December 31, 2019 were obtained from the hospitalization data provided by the Hospital Authority (HA). Cases were defined by having a principal diagnosis of acute appendicitis, corresponding to the diagnosis code of 540.x in the International Classification of Diseases, Nineth Revision, Clinical Modification (ICD-9-CM).

Weekly meteorological data, including mean temperature, mean relative humidity and total rainfall were obtained from the Hong Kong Observatory (HKO). Among the 86 HKO monitoring stations, temperature, humidity and rainfall data were available from 49, 35 and 51 stations respectively, covering all 18 districts in Hong Kong. The data obtained were considered representative of the overall meteorological conditions of Hong Kong.

The weekly influenza rates in Hong Kong were estimated by a composite metric called the influenza-like illness positive (ILI+) rate. Due to the nature of influenza infection, the majority of cases do not result in hospital admission and hence hospitalization data is not an appropriate measure of infection rate. Instead, the rate can be estimated from the weekly number of consultations for influenza-like illness (ILI) recorded by a local sentinel surveillance system consisting of HA’s general outpatient clinics and a number of private clinics [11, 12]. The surveillance system defines ILIs based on symptoms, including fever, cough and sore throat [11, 12]. To obtain a more reliable estimate of influenza infection rates with the input of laboratory data, past studies have used a composite index, ILI + rate, which adjusts the incidence of ILI by the proportion of respiratory specimens from ILI patients tested positive for particular strains of influenza virus (A/H1N1, A/H3N2, B) [12]. The use of this index helps minimize the problem of overdiagnosis of influenza. Symbolically, the ILI + index for a particular influenza strain is calculated by:

$$\text{ILI consultations}\times\frac{\text{number of respiratory specimens tested positive for that strain}}{\text{total number of respiratory specimens tested}}$$

Some studies have suggested that the levels of certain air pollutants may be positively associated with the incidence of acute appendicitis [13, 14]. In this study, the effect of air pollution were included in the statistical model for confounder control. The weekly mean atmospheric concentrations of ozone (O3), nitrogen dioxide (NO2), sulfur dioxide (SO2) and fine particulate matter (PM_2.5_) were obtained from the Environmental Protection Department (EPD) of Hong Kong. Since the levels of these pollutants were highly correlated with each other, and the impacts of individual pollutants were not the interest of this study, a composite index (referred to as “air pollution health risk index” below) was calculated as a summary measure of air pollution, and treated as a single variable in the statistical model. The index was adopted, with slight modification, from the Air Quality Health Index (AQHI) developed by Wong et al. and currently used by the EPD to inform the public of air pollution levels in the city [15, 16]. The index was designed to reflect the percentage added health risk (%AR) due to various air pollutants. The version of the index used in this study was calculated by:

*Air Pollution Health Risk Index = %AR(O*_*3*_*) + %AR(NO*_*2*_*) + %AR(SO*_*2*_*) + %AR(PM*_*2.5*_*)*, where *%AR(pollutant*_*i*_*) = [exp(β*_*i*_ *× pollutant*_*i*_*) − 1] × 100%*. The β values (×10^− 4^) for O_3_, NO_2_, SO_2_ and PM_2.5_ were 5.116328, 4.462559, 1.393235 and 2.180567 respectively. The concentration of air pollutants was measured in µg/m^3^.

### Statistical model

The model used in this study is a combination of the generalized additive model (GAM) with quasi-Poisson distribution and the distributed-lag non-linear model (DLNM). GAM is a flexible non-parametric model that makes use of scatterplot smoothers [17]. Due to its non-parametric nature, it is not limited by assumptions such as linearity and monotonicity. On the other hand, DLNM is ideal for modelling the delayed effects of environmental exposures on health outcomes, when their correlations are nonlinear in nature [18]. GAM with DLNM has been adopted in a number of time-series ecological studies similar to this study, and was able to generate meaningful results [12, 19, 20]. 

Symbolically, the model of this study is:

*log(µ*_*t*_*) = intercept + cb(temp*_*t*_; *lag) + cb(humid*_*t*_; *lag) + cb(rain*_*t*_; *lag) + cb(pollution*_*t*_; *lag) + cb(ILI_positive_A/H1N1*_*t*_; *lag) + cb(ILI_positive_A/H3N2*_*t*_; *lag) + cb(ILI_positive_B*_*t*_; *lag) + s(year*_*t*_*) + s(week*_*t*_*) + offset*_*t*_.

where the dependent variable *µ*_*t*_ is the expected number of hospital admissions due to acute appendicitis in week *t*. The mean temperature, mean relative humidity and total rainfall in week *t* are denoted as *temp*_*t*_, *humid*_*t*_ and *rain*_*t*_ respectively. The air pollution health risk index in week *t* is denoted as *pollution*_*t*_. The influenza-like illness positive (ILI+) rates specific to strains A/H1N1, A/H3N2 and B in week *t* are denoted as *ILI_positive_A/H1N1*_*t*_, *ILI_positive_A/H3N2*_*t*_ and *ILI_positive_B*_*t*_ respectively. The function *cb(.)* is a cross-basis function for modeling the exposure-response relationship after taking lag-effects into consideration, and the maximum lag period is 2 weeks. The function *s(.)* is a spline function for smoothing. The terms *year*_*t*_ and *week*_*t*_ are included to control for long-term trends and seasonal patterns respectively. The term *offset*_*t*_ represents the number of all-cause hospitalizations in week *t*. The degrees of freedom were chosen based on the principles of minimizing the generalized cross validation score and maintaining simplicity of the model.

The strength of association is reported in the form of cumulative adjusted relative risks (with the corresponding 95% confidence intervals). The reference values of the ILI + rates and rainfall are set at zero, while those of temperature, relative humidity and the air pollution health risk index are set at their respective medians.

### Sensitivity analyses

Four separate sensitivity analyses were performed to assess the robustness of the model. The first analysis was carried out by excluding the data from 2009, when there was an unusual ILI + pattern due to the novel H1N1 (swine flu) pandemic. The second analysis was carried out by altering the degrees of freedom of the cross-basis function in the model. The third analysis was done by halving and doubling the lag period to 1 week and 4 weeks respectively. The fourth analysis used each of the individual air pollutants in lieu of the composite air pollution health risk index to construct separate models for examination.

### Software for analysis

Data management and statistical analyses were performed in the R programming environment (version 4.3.2), with the packages “dplyr”, “mgcv” and “dlnm”.

## Results

### Descriptive statistics

**Fig. 1:**
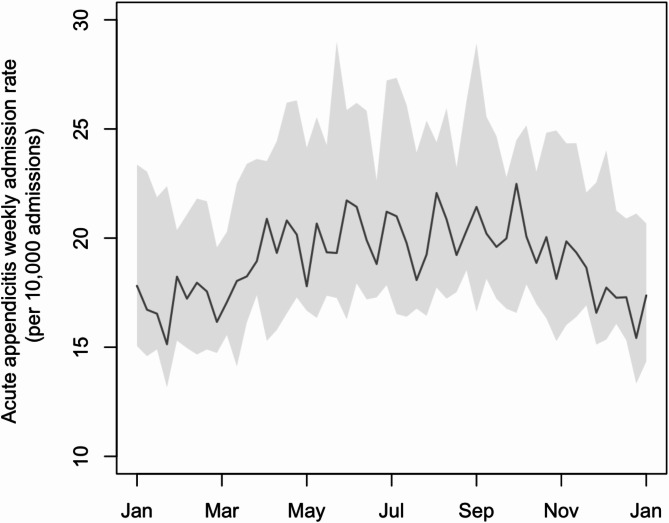
Seasonal trend of weekly acute appendicitis admission rate from 1998 to 2019 The central line and the grey bands represent the median and interquartile ranges respectively

Weekly data of acute appendicitis admissions and total admissions in public hospitals, meteorological variables, air pollutant levels and ILI + indices for types A/H1N1, A/H3N2 and B from years 1998 to 2019 were collected. In this 22-year period, there were 59,128 cases of acute appendicitis in total. The median weekly admission rate of acute appendicitis was 19.05 cases per 10,000 admissions. There was a seasonal pattern of acute appendicitis rate, with summer having a higher rate in general (Fig. 1).

During the study period, the medians of weekly mean temperature, mean relative humidity and total rainfall were 24.61 °C, 79.64% and 11.40 mm respectively. The median of the air pollution health risk index was 5.26. The medians of the weekly ILI + indices for influenza types A/H1N1, A/H3N2 and B were 2.21, 9.73 and 4.49 per 10,000 consultations respectively. Both types A/H1N1 and B showed seasonal trends with a single peak at winter/spring, while type A/H3N2 showed a seasonal trend with two peaks at winter/spring and summer (Figure S1).

Table 1 shows the descriptive statistics of the aforementioned variables in detail. A correlation matrix for the covariates, using Kendall’s tau-b (τ_B_) coefficients, is available in Table S1. It was found that moderate correlations exist between mean relative humidity and total rainfall (τ_B_ = 0.464), and between mean relative humidity and air pollution level (τ_B_ = − 0.407).

**Table 1:** Descriptive statistics of weekly acute appendicitis admission rate, meteorological variables, air pollution level and influenza activity from 1998 to 2019

	5th Percentile	25th Percentile	Median	75th Percentile	95th Percentile
Weekly admission rate of acute appendicitis (per 10,000 admissions)	12.76	15.94	19.05	23.87	31.91
Meteorological variables					
Weekly mean temperature (°C)	15.07	19.51	24.61	27.97	29.69
Weekly mean relative humidity (%)	62.76	74.43	79.64	83.57	88.69
Weekly total rainfall (mm)	0.00	0.30	11.40	57.45	200.89
Air pollution					
Air pollution health risk index*	2.90	4.13	5.26	6.37	7.99
Influenza activity (ILI+: influenza-like illness-positive index, per 10,000 consultations)					
ILI + A/H1N1	0.00	0.00	2.21	10.70	70.63
ILI + A/H3N2	0.00	2.76	9.73	37.29	140.19
ILI + B	0.00	1.46	4.49	13.88	50.81

*Calculated based on the weekly mean concentrations of atmospheric ozone (O_3_), nitrogen dioxide (NO_2_), sulfur dioxide (SO_2_) and fine particulate matter (PM_2.5_), to indicate the overall health risk attributable to air pollution

### Results of primary analysis

**Fig. 2:**
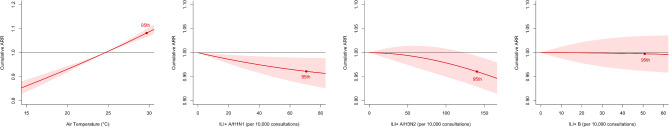
Cumulative adjusted relative risks (ARRs) of acute appendicitis admission against the major independent variables of interest The central line and the red bands represent the point estimates and 95% confidence intervals of the cumulative ARRs respectively. The red dots labelled “95th ” indicate the ARRs at the 95th percentiles of the independent variables

Among the independent variables of interest, it was found that high temperature was significantly associated with a higher risk of acute appendicitis admission. The cumulative adjusted relative risk (ARR) comparing the 95th percentile with the median temperature was 1.082 (95% confidence interval, CI: 1.065–1.099). In contrast, both ILI + A/H1N1 and ILI + A/H3N2 showed significant negative associations with acute appendicitis admission. The cumulative ARRs comparing the 95th percentiles with zero for ILI + A/H1N1 and ILI + A/H3N2 were 0.961 (95% CI: 0.934–0.989) and 0.961 (95% CI: 0.929–0.993) respectively. ILI + B, on the other hand, showed no significant association with acute appendicitis admission. The cumulative ARR comparing the 95th percentile with zero for ILI + B was 0.998 (95% CI: 0.962–1.035). Figure 2 displays graphically the cumulative ARRs (with 95% CIs) of the above 4 variables at different levels, while Table 2 displays the ARRs at their 95th percentiles at different lag times.

**Table 2:** Cumulative adjusted relative risks (ARRs) of major independent variables of interest at their 95th percentiles at different lag times

	Weekly mean temperature	ILI + A/H1N1	ILI + A/H3N2	ILI + B
Reference level	Median	0	0	0
Adjusted relative risk (95% confidence interval) at lag:				
Lag zero	1.060* (1.042–1.090)	1.003 (0.956–1.053)	0.980 (0.921–1.041)	0.997 (0.936–1.060)
Lag at 1st week	1.030* (1.021–1.030)	0.987* (0.977–0.996)	0.987* (0.976–0.998)	0.999 (0.987–1.010)
Lag at 2nd week	0.990 (0.966–1.010)	0.971 (0.925–1.019)	0.994 (0.936–1.056)	1.001 (0.941–1.060)
Cumulative	1.082* (1.065–1.099)	0.961* (0.934–0.989)	0.961* (0.929–0.993)	0.998 (0.962–1.035)

*Statistically significant

**Fig. 3:**
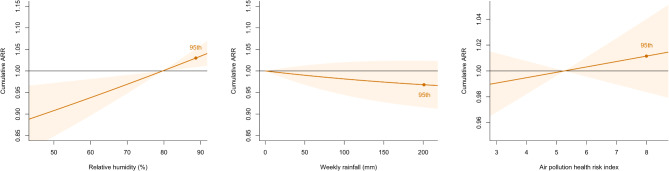
Cumulative adjusted relative risks (ARRs) of acute appendicitis admission against the other independent variables in the model The central line and the orange bands represent the point estimates and 95% confidence intervals of the cumulative ARRs respectively. The orange dots labelled “95th ” indicate the ARRs at the 95th percentiles of the independent variables

Among the other independent variables, relative humidity showed significant positive association with acute appendicitis admission. The cumulative ARR comparing the 95th percentile with the median relative humidity was 1.030 (95% CI: 1.009–1.052). Rainfall showed a negative but insignificant association with acute appendicitis admission. The cumulative ARR comparing the 95th percentile with zero was 0.968 (95% CI: 0.915–1.023). The air pollution health risk index was positively but insignificantly associated with acute appendicitis admission. The cumulative ARR comparing the 95th percentile with the median value was 1.011 (95% CI: 0.984–1.040). Figure 3 displays graphically the cumulative ARRs (with 95% CIs) of these variables at different levels.

### Results of sensitivity analyses

In the first sensitivity analysis (analysis A in Table S2), data from year 2009 were removed. It was found that the association between ILI + A/H1N1 and acute appendicitis admission became marginally insignificant (cumulative ARR at 95th percentile: 0.966, 95% CI: 0.929–1.004). The cumulative ARRs of temperature and ILI + A/H3N2 remained significant, and that of ILI + B remained insignificant.

In the second sensitivity analysis (analysis B in Table S2), the degrees of freedom of the predictor-response matrix and lag-response matrix in the cross-basis functions were changed from 2 to 4, while the data were not altered. It was found that the cumulative ARRs of temperature, ILI + A/H1N1 and ILI + A/H3N2 all remained significant, and that of ILI + B remained insignificant.

In the third sensitivity analysis (analysis C in Table S2), the lag period was changed from 2 weeks to 1 week and 4 weeks respectively. In both cases, the cumulative ARRs of temperature, ILI + A/H1N1 and ILI + A/H3N2 all remained significant, and that of ILI + B remained insignificant.

In the fourth sensitivity analysis (analysis D in Table S2), each of the individual air pollutants was used to construct separate models instead of the composite air pollution health risk index. In all 4 single-pollutant models, the cumulative ARRs of temperature, ILI + A/H1N1 and ILI + A/H3N2 all remained significant, and that of ILI + B remained insignificant.

## Discussion

This study is the first ecological study on acute appendicitis incidence that incorporates meteorological factors, strain-specific influenza activity, and air pollution data into the same statistical model, allowing for detailed multivariate analysis and comprehensive confounder control. The use of weekly data also ensured a high temporal resolution and enabled the model to control for seasonal patterns.

The positive association between ambient temperature and acute appendicitis admission found in this study is consistent with previous studies [6–8]. Some postulate that the effect of high temperature on the pathogenesis of acute appendicitis may be mediated by the sequence of dehydration and constipation [8]. It is know that impaction of hardened feces in the appendix (formation of appendicolith) can lead to luminal obstruction and predispose to appendicitis [4]. Exposure to high temperature can lead to excessive sweating and respiratory water loss, hence causing dehydration. This in turn increases the risk of constipation and formation of hardened feces around the appendix, leading to a higher chance of developing acute appendicitis.

Regarding the relationship between influenza activity and acute appendicitis admission, this study found that high ILI + rates for both types A/H1N1 and A/H3N2 are associated with a lower risk of acute appendicitis admission. This result appears to deviate from the positive association between annual influenza and appendicitis rates found in the previous study by Alder et al. [9] However, it is important to note that Alder et al. used yearly data for analysis, while this study used weekly data, providing a much higher resolution in the time dimension and allowing the correction of seasonality effect. Moreover, meteorological factors and air pollution were adjusted for in this study, but not in that by Alder et al. Therefore, it can be contended that the independent effect of influenza A infection may have been better isolated in this study.

A possible explanation for the apparent negative association between type A influenza and acute appendicitis admission found in this study is that personal hygiene measures, such as hand washing and mask wearing, were employed more meticulously during the peaks of seasonal influenza. This might have caused reduced transmission of other viruses, such as adenovirus, cytomegalovirus and Epstein-Barr virus, which are known to cause certain variants of acute appendicitis [21]. Such behavioral changes in response to influenza season are plausible, as demonstrated in a previous study revealing a 17% increase in odds in hand hygiene adherence during influenza periods [22]. 

On the other hand, influenza type B is typically perceived by public as less health-threatening than type A [23]. Thus, even during the seasonal peak of influenza type B, the public’s awareness in personal hygiene may not be particularly high. As a result, transmission of appendicitis-related viruses was not affected. This is consistent with the result that ILI + B was not significantly associated with acute appendicitis admission.

Another potential explanation for the negative association between type A influenza and acute appendicitis admission is the phenomenon of viral interference. It is known that infection by one virus can sometimes lead to a protection effect against the infection of another unrelated virus, through the activation of nonspecific host immune response such as release of cytokines [24]. It is conceivable that the infection by type A influenza may temporarily reduce the risk of infection by appendicitis-related viruses through this mechanism. Epidemiological data in Hong Kong also support the existence of interference between influenza and other respiratory viruses, such as adenovirus, in the region [25]. Nonetheless, existing literatures focus on the effects of viral interference in the respiratory tract. Further study is needed to establish and characterize such effects in the digestive system, and their relationship with the pathogenesis of acute appendicitis.

In the sensitivity analysis, where the data from 2009 were excluded, the association with influenza A/H1N1 became marginally insignificant (95% CI: 0.929–1.004). This indicates that health behaviors and other lifestyle changes during times of epidemics may play a role in the observed negative association between influenza A/H1N1. Nonetheless, the effect estimate still favors the negative association when data from 2009 was removed.

Among the other variables, relative humidity was shown to positively and significantly associate with acute appendicitis admission. It is conceivable that when humidity is high, sweat evaporates more slowly and therefore sweating is less effective in cooling our bodies. In other words, high humidity may magnify the impact of high ambient temperature on the risk of acute appendicitis. In addition, some researchers suggest that transient exposure to high humidity may affect the microbiota composition in the guts [26]. This may have a role in contributing to the development of acute appendicitis.

While this study provided evidence on the potential impact of meteorological factors and influenza activity on the risk of acute appendicitis, it has several limitations. Firstly, stratified data of acute appendicitis admission by sex and age groups were not available, and therefore subgroup analysis could not be performed. Secondly, weekly influenza vaccination rates were not available, and were not controlled for in the model. Vaccination does not only reduce the risk of seasonal influenza infection, but also alters the clinical presentation in those infected. Thus, vaccination rate is a potentially important confounder and effect modifier that the model of this study could not take into account. Thirdly, due to the nature of using aggregated data, it was impossible to ascertain the exposures and outcome in the individual level. For instance, the meteorological variables only represented the average weather condition in Hong Kong. Different individuals live and work in different districts and have varying lifestyles, which cause their individual exposures to differ from the territory-average values. Lastly, systematic changes during the 22 years of observation could not be controlled for, such as population mobility, overall change in healthcare-seeking behavior and alterations in diagnostic methods in clinical practice. We acknowledge that the above limitations may impact the robustness of the findings of our study. Nevertheless, this study paved the way for further investigation using studies with individual-level data and more sophisticated design.

## Conclusion

This study found a positive association between ambient temperature and acute appendicitis admission. Our findings suggest that healthcare institutions are likely to experience surges in hospital admissions during hot weather, underscoring the need for improved resource planning, staffing, and emergency response. In addition, we found a negative association between infection of influenza type A (both H1N1 and H3N2) and acute appendicitis admission, warranting further investigations to validate the findings.

## Electronic supplementary material

Below is the link to the electronic supplementary material.

Supplementary Material 1

Supplementary Material 2

## Data Availability

The data that support the findings of this study are available from the Hospital Authority, The Government of the Hong Kong Special Administrative Region but restrictions apply to the availability of these data, which were used under license for the current study, and so are not publicly available.
