# Supplementary material for: Association of ambient temperature and influenza-like illness with acute appendicitis: an ecological study using 22-year data
Source: BMC Public Health. 2025 Mar 29;25:1191. doi: 10.1186/s12889-025-22318-x (PMC11954316; doi:10.1186/s12889-025-22318-x)
Supplement: Supplementary file 1 — Supplementary Material 1 [file 12889_2025_22318_MOESM1_ESM.docx]

**Table S1**. Correlation matrix of covariates, using Kendall's tau-b (τ_B_) coefficients

|  | **Temp** | **Humidity** | **Rainfall** | **Pollution** | **ILI+ H1N1** | **ILI+ H3N2** |
| --- | --- | --- | --- | --- | --- | --- |
| **Temp** | / |  |  |  |  |  |
| **Humidity** | 0.103 | / |  |  |  |  |
| **Rainfall** | 0.286 | 0.464 | / |  |  |  |
| **Pollution** | -0.304 | -0.407 | -0.385 | / |  |  |
| **ILI+ H1N1** | -0.110 | 0.105 | 0.016 | -0.038 | / |  |
| **ILI+ H3N2** | 0.090 | 0.113 | 0.096 | -0.123 | -0.076 | / |
| **ILI+ B** | -0.086 | 0.132 | 0.019 | -0.051 | 0.192 | 0.109 |

Temp: weekly mean temperature; Humidity: weekly mean relative humidity; Rainfall: weekly total rainfall; Pollution: air pollution health risk index; ILI+ H1N1: ILI+ rate for influenza A, strain H1N1; ILI+ H3N2: ILI+ rate for influenza A, strain H3N2; ILI+ B: ILI+ rate for influenza B

|  | Primary analysis | Sensitivity analysis A | Sensitivity Analysis B | Sensitivity Analysis C | Sensitivity Analysis D |
| --- | --- | --- | --- | --- | --- |
| Change from primary analysis | N/A | Removing data from year 2009 | Changing the degrees of freedom of the predictor-response matrix and lag-response matrix to 4 | Changing the lag period to 1 and 4 | Using individual air pollutants as covariates (separate models) instead of air pollution index |
| Cumulative adjusted relative risk (95% confidence interval) at the 95^th^ percentile [* = statistically significant] | | | | | |
| Temperature | 1.082*  (1.065 – 1.099) | 1.079*  (1.062 – 1.096) | 1.086*  (1.069 – 1.104) | Lag 1: 1.086*  (1.071 – 1.100)  Lag 4: 1.086*  (1.065 – 1.108) | NO_2_-only: 1.080* (1.061 – 1.099)  SO_2_-only: 1.079* (1.063 – 1.095)  O_3_-only: 1.078* (1.063 – 1.095)  PM_2.5_-only: 1.078* (1.060 – 1.096) |
| ILI+ A/H1N1 | 0.961*  (0.934 – 0.989) | 0.966  (0.929 – 1.004) | 0.965*  (0.936 – 0.995) | Lag 1: 0.965*  (0.939 – 0.992)  Lag 4: 0.965*  (0.935 – 0.996) | NO_2_-only: 0.961* (0.933 – 0.989)  SO_2_-only: 0.960* (0.933 – 0.988)  O_3_-only: 0.961* (0.933 – 0.989)  PM_2.5_-only: 0.960* (0.933 – 0.988) |
| ILI+ A/H3N2 | 0.961*  (0.929 – 0.993) | 0.957*  (0.925 – 0.990) | 0.961*  (0.929 – 0.994) | Lag 1: 0.964*  (0.933 – 0.995)  Lag 4: 0.957*  (0.925 – 0.991) | NO_2_-only: 0.960* (0.929 – 0.993)  SO_2_-only: 0.960* (0.929 – 0.992)  O_3_-only: 0.961* (0.929 – 0.993)  PM_2.5_-only: 0.958* (0.927 – 0.991) |
| ILI+ B | 0.998  (0.962 – 1.035) | 0.997  (0.958 – 1.037) | 1.002  (0.966 – 1.040) | Lag 1: 0.997  (0.962 – 1.033)  Lag 4: 1.003  (0.965 – 1.042) | NO_2_-only: 0.998 (0.962 – 1.035)  SO_2_-only: 0.997 (0.961 – 1.034)  O_3_-only: 0.996 (0.960 – 1.033)  PM_2.5_-only: 0.999 (0.963 – 1.036) |

**Table S2**. Sensitivity analyses
