# Supplementary material for: Association of ambient temperature and influenza-like illness with acute appendicitis: an ecological study using 22-year data
Source: BMC Public Health. 2025 Mar 29;25:1191. doi: 10.1186/s12889-025-22318-x (PMC11954316; doi:10.1186/s12889-025-22318-x)
Supplement: Supplementary file 2 — Supplementary Material 2 [file 12889_2025_22318_MOESM2_ESM.pdf]

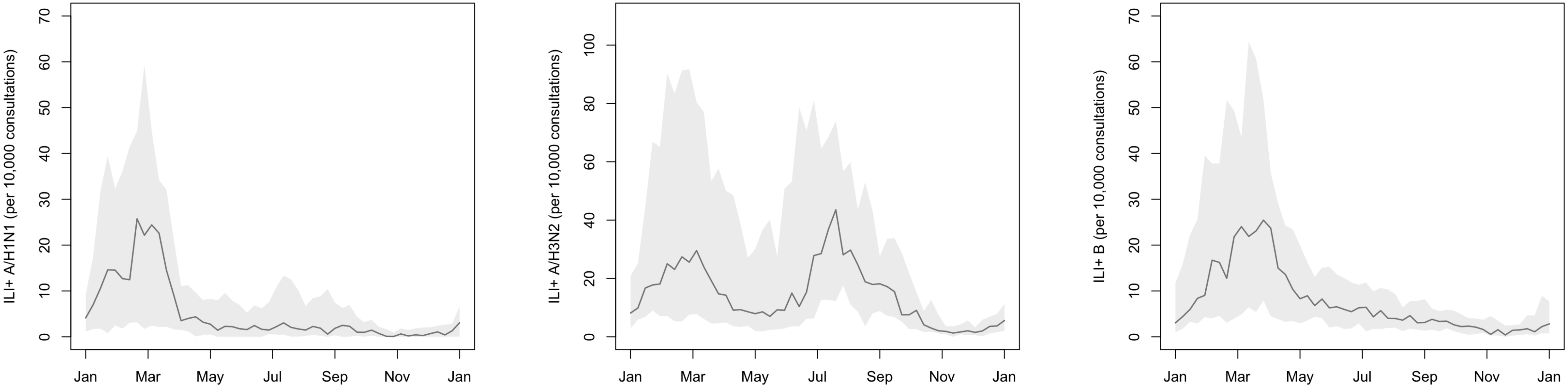

Figure S1. Seasonal trend of weekly ILI+ rates from 1998 to 2019. The central lines and the grey bands represent the medians and interquartile ranges respectively.
